# Facilitators and Barriers to Safe Medication Administration to Hospital Inpatients: A Mixed Methods Study of Nurses’ Medication Administration Processes and Systems (the MAPS Study)

**DOI:** 10.1371/journal.pone.0128958

**Published:** 2015-06-22

**Authors:** Monsey McLeod, Nicholas Barber, Bryony Dean Franklin

**Affiliations:** 1 The Centre for Medication Safety and Service Quality, Pharmacy Department, Imperial College Healthcare NHS Trust, London, United Kingdom, and the Research Department of Practice and Policy, UCL School of Pharmacy, London, United Kingdom; 2 The Health Foundation, London, United Kingdom; Örebro University, SWEDEN

## Abstract

**Context:**

Research has documented the problem of medication administration errors and their causes. However, little is known about how nurses administer medications safely or how existing systems facilitate or hinder medication administration; this represents a missed opportunity for implementation of practical, effective, and low-cost strategies to increase safety.

**Aim:**

To identify system factors that facilitate and/or hinder successful medication administration focused on three inter-related areas: nurse practices and workarounds, workflow, and interruptions and distractions.

**Methods:**

We used a mixed-methods ethnographic approach involving observational fieldwork, field notes, participant narratives, photographs, and spaghetti diagrams to identify system factors that facilitate and/or hinder successful medication administration in three inpatient wards, each from a different English NHS trust. We supplemented this with quantitative data on interruptions and distractions among other established medication safety measures.

**Findings:**

Overall, 43 nurses on 56 drug rounds were observed. We identified a median of 5.5 interruptions and 9.6 distractions per hour. We identified three interlinked themes that facilitated successful medication administration in some situations but which also acted as barriers in others: (1) system configurations and features, (2) behaviour types among nurses, and (3) patient interactions. Some system configurations and features acted as a physical constraint for parts of the drug round, however some system effects were partly dependent on nurses’ inherent behaviour; we grouped these behaviours into ‘task focused’, and ‘patient-interaction focused’. The former contributed to a more streamlined workflow with fewer interruptions while the latter seemed to empower patients to act as a defence barrier against medication errors by being: (1) an active resource of information, (2) a passive information resource, and/or (3) a ‘double-checker’.

**Conclusions:**

We have identified practical examples of system effects on work optimisation and nurse behaviours that potentially increase medication safety, and conceptualized ways in which patient involvement can increase medication safety in hospitals.

## Introduction

Medication administration errors (MAEs) occur in 8.0% to 19.6% of doses in hospitals worldwide [1]. Although these figures should be interpreted with some caution due to important differences among studies [2], it is clear that MAEs are common. Even in countries where MAE rates appear relatively low, such as the United Kingdom (5.6% of non-intravenous doses administered to adult hospital inpatients [2]), it has been estimated that 0.6–21% of MAEs may lead to severe patient harm [3]. Considering the vast number of medication administrations that occur, the actual number of patients that suffer harm is likely to be substantial. However, while many studies have measured the incidence of MAEs [1,2] and identified causes that arise from both individuals and systems [4], few have examined this area from the other perspective: how do nurses work within hospital systems to administer medications safely and successfully?

A system is any set of interdependent elements or processes interacting to achieve a common aim [5]. High-profile public inquiries and reports [5–11] provide a stark reminder that while humans err, systems can fail. In some cases, system failures that contributed to patient suffering were also the result of an organizational culture that disproportionately prioritized achieving financial targets over providing quality patient care [12]. System-based failures in healthcare organizations can occur in any processes and are likely to be important underlying contributory factors for recurring medication errors [5,10,13]. Poorly designed systems and overly complicated processes can increase the risk of an error occurring, while intuitive user-centred designed systems and more streamlined or simpler processes may reduce this risk [13,14].

In the context of hospital drug administration rounds, persistent system-related challenges such as medicines not being available, limited equipment, inefficient workflow and frequent interruptions are known contributors to MAEs [15,16]. However, variation in ward-based systems (such as medication ordering, storage and transport systems) exist within and between countries [3,17,18]; research suggests such variation can affect the frequency of different types of MAEs [19–22]. While it is important that such causes of MAEs are identified, this only provides us with information about which systems and processes do not work well. It does not tell us which *do* work well. For instance, studies of reworks and workarounds associated with medication administration suggest that alternative and sometimes ‘deviant’ processes (which may also be procedural failures, violations, shortcuts or improvisations) are relatively common and can create ‘more holes in the system’, bypassing essential safety defence barriers, and thereby increasing the risk of a medication safety incident occurring [23–26]. However, in some cases, alternative or deviant processes may be deliberate pre-emptive actions taken by an individual to increase efficiency or to overcome known error-prone system-based failures [25]. Thus, alternative processes may act as an indicator of underlying latent conditions for future incidents [13]. It is therefore important to not only identify potential contributory factors for MAEs but also how individuals manage them within the resources available.

We aimed to address this knowledge gap by identifying and describing system factors that facilitate and/or hinder successful medication administration, focused on three interrelated areas: (1) individual nurse practices and workarounds, (2) medication administration workflow, and (3) the frequency and nature of interruptions and distractions during medication administration.

## Methods

This was a mixed methods ethnographic study of medication administration by nursing staff in three wards, each in a different English National Health Service (NHS) hospital trust. Study wards were purposively selected to represent a range of inpatient medication systems; the sampling frame was based on findings from our national survey of hospital medication systems [27]. One ward (site A) was selected as a ‘typical’ English inpatient ward (including paper medication prescription charts, patient bedside medication lockers plus drug trolleys, and the use of patients’ own medications where appropriate). One ward (site B) used an established electronic prescribing and medication administration (EPMA) system (since 2008). The final ward (site C) used a relatively new EPMA system (since July 2012) and had two nurses administering medication together to each patient. Other ward characteristics and dates of observation are summarised in Table 1.

**Table 1:** Characteristics of study wards and summary of data collected.

Study wards	Staffing	Medication systems and administration processes	Observations
**Site A**: 27-bed vascular/ cardiology ward in an acute NHS trust	24 nurses. Observed nurse to patient ratio 1:8 on both day shift and night shift. Nurse participants reported fewer staff than usual during the data collection period	Paper drug prescription and administration chart; four drug trolleys; RFID controlled electronic bedside medication cabinets; nurses administered drugs to patients they were looking after; patient’s own drugs from home were permitted to be used during their inpatient stay.	26 March to 3 April 2012; 14 nurses (includes 2 bank/agency)^a^ (13 female; 1 male); 18 drug rounds (three at 6am and five each at 12pm, 6pm and 10pm); total 27 hours of observation, of which 15 hours 20 min were during drug rounds; 11 hours 40 min were before and after drug rounds.
**Site B**: 28-bed adult elective surgical ward in an acute hospital of a foundation NHS trust	16 nurses. Observed nurse to patient ratio 1:6 on both day shift and night shift. Nurse participants reported fewer patients than usual during the data collection period	Trust-wide EPMA system since 2008; EPMA access: two desktop computers, three tablet devices, and one COW; two drug trolleys; RFID controlled bedside medication cabinets; nurses administered drugs to patients they were looking after; patient’s own drugs from home were permitted to be used during their inpatient stay.	20–31 August 2012; 13 nurses (includes 2 bank/agency)^a^ (12 female; 1 male); 20 drug rounds (four at 6am, five at 12pm, six at 6pm, and five at 10pm); total 29 hours of observation, of which 14 hours 13 min were during drug rounds; 14 hours 47 min were before and after drug rounds.
**Site C**: 18-bed adult neurological rehabilitation ward in an acute hospital of a foundation NHS trust	15 nurses. Observed nurse to patient ratio 1:9 on both day shift and night shift. Nurse participants reported fewer staff than normal during the data collection period	EPMA system since July 2012, trust-wide roll out in progress at time of data collection; EPMA access: one desktop computer, one laptop attached to the drug trolley, and two COWs; one large drug trolley; conventional metal bedside medication lockers; two nurses administered drugs to all patients together; ‘opt-out’ patient self-administration policy; patients’ own drugs from home were permitted to be used during their inpatient stay; HCAs helped patients to take their medications after the nurse had prepared the doses.	12–19 November 2012; 16 nurses (includes 3 bank/agency)^a^ (13 female; 3 male); 18 drug rounds (two at 6am, four at 8am, four at 12pm, five at 6pm, and three at 10pm); total 29 hours of observation, of which 20 hours 35 min were during drug rounds; 8 hours 25 min were before and after drug rounds; no IV doses were prescribed (patients on this ward do not usually require IVs).

Abbreviations: COW, computer on wheels; EPMA, electronic prescribing and medication administration system; HCA, health care assistant; IV, intravenous; NHS, National Health Service; RFID, radio frequency identification.

^a^Bank nurses were employees of the hospital trust who may also be a regular staff member of the study ward. Agency nurses were employees of an external company who were contracted by the hospital trust to provide nursing staff cover to wards for specific work shifts.

### Ethics statement

NHS research ethics approval was not required as this study met the criteria for service evaluation and focused on staff as participants [28]. Academic research ethics approval was granted by the School of Pharmacy, University of London, in January 2011. All participants provided written consent.

### Data collection

Nurses mainly administered medications during scheduled drug rounds (Table 1); data collection therefore focused around these times. A convenience sample of nurses was observed by one experienced pharmacist researcher (MM) for seven to ten consecutive days on each ward. Prior to starting observations, MM went through a participant information leaflet with the nurse concerned, answered any questions and requested written consent. Observations were divided into ‘qualitative’ and ‘quantitative’: each was conducted during separate drug rounds. MM observed nursing staff as they went about their usual routines before, during and after scheduled rounds. The start time, duration, number of patients, and number of steps taken by the nurse (using a pedometer, Yamax Digi-Walker SW-200) during each observed drug round were documented. During the first set of observed drug rounds (qualitative), detailed descriptions of the medication administration processes and systems were documented as field notes, photographs, ‘spaghetti diagrams’ (maps of the ward annotated by hand to show nurses’ walking patterns during drug rounds), and narratives. Field notes ranged from including the greatest level of detail, described by Lofland and Lofland [29] as ‘practices’ (an activity that the participants regard as unremarkable normal feature of on-going life), to the highest level ‘lifestyles or subcultures’ (the global adjustments to life by large numbers of similarly situated persons). The level of detail to be abstracted during observations was not determined *a priori* as we wished to explore interactions at different levels between ‘humans’ and ‘systems’. During the second set of observed drug rounds (quantitative), additional quantitative data plus explanatory field notes were documented: details of the medicines administered, storage locations accessed, and the number and sources of interruptions and distractions. Data collection was not focused on detecting MAEs; however the number of observed opportunities for error (as defined below) was documented so that the MAE rate could also be calculated if any MAEs were observed.

### Definitions and categories

To facilitate interpretation of our quantitative findings [2] we used Allan and Barker’s [30] MAE definition: a deviation from the prescriber’s medication order as written on the patient’s chart. Errors prevented by the patient or persons other than the nurse themselves were also included as MAEs. The following were excluded as MAEs: wrong time, doses omitted for therapeutic reasons or due to the patient not being on the ward, and procedural violations such as not checking the patient’s allergy status or leaving a dose at the patient’s bedside for the patient to self-administer. All doses where both preparation and administration were observed were included as opportunities for error (OE); the total number of OEs was the denominator for calculating MAE rates. Detailed definitions of these are provided elsewhere [3].

There is no standard operational definition for an interruption or distraction, or standard categories for the source of the interruption or distraction [31]. We therefore adapted previous definitions [16,31,32]: an interruption was defined as a situation in which a nurse ceased the medication preparation, administration and/or documentation task before it was complete [31,32] and a distraction was a stimulus from a source external to the nurse that was not followed by cessation of activity but by the nurse continuing productive efforts while responding in an observable manner [32]. Specific sources of interruptions and distractions were grouped into 16 categories [31,33]; detailed definitions of the sources of interruptions and distractions are reported elsewhere [3].

### Data analysis

All data were transcribed within 24 hours of observation to maximize recall, identify further areas to focus subsequent observations, and facilitate concomitant data analysis during data collection. Qualitative data were analysed using a framework approach [34]; an initial high level thematic framework based on the study objectives was produced which comprised: medication systems available, use of medication systems in practice, medication safety, drug round workflow, and interruptions and distractions. MM analysed all data and further developed the framework; BDF independently reviewed several iterations of the expanded thematic framework, verified the coding schemes and indexed two sets of field notes from each study ward (approximately 10% of all field notes). The final coding scheme and thematic framework were produced after further iterative work by MM and BDF. Quantitative data were summarized descriptively. MAE rates were calculated for both non-IV doses and IV doses [2] as a percentage of the total number of OEs observed. Separate and combined interruption and distraction rates per drug round hour were also calculated [31,35].

### Authenticity, plausibility, and criticality

We used three interpretative criteria to increase validity of our findings: authenticity, plausibility, and criticality [36]. Authenticity has been described as “immersion in the case through extended fieldwork” [36]; we report in our findings a range of evidence to demonstrate this. Plausibility is “developing explanations of local phenomena which made sense to participants and drawing these together into a coherent overall narrative” [36]; this was achieved through feedback and discussions with participants at the end of the observation period at each site. Criticality is the systematic questioning of assumptions made in describing the explanations of the phenomena under study. Both plausibility and criticality considerations also formed a key component of the data analysis by having two researchers (MM and BDF) analyse the data iteratively until both agreed on the final themes.

## Results

Overall, 85 hours and 43 different nurses on 56 drug rounds (26 qualitative and 30 quantitative) were observed across the three study wards (Table 1). One newly qualified nurse who initially declined later agreed to be observed when she was able to give medications unsupervised. All other nurses consented to participate. Verbal nursing feedback suggested that they did not mind being observed and many expressed interest in the study.

During the quantitative observations, 458 doses were included as OEs (445 non-IV and 13 IV doses). The MAE rates were 2.7% of non-IV OEs (95% confidence interval (CI), 1.2 to 4.2) and 30.8% of IV OEs (95% CI, 26.3 to 35.2).

**Fig 1:**
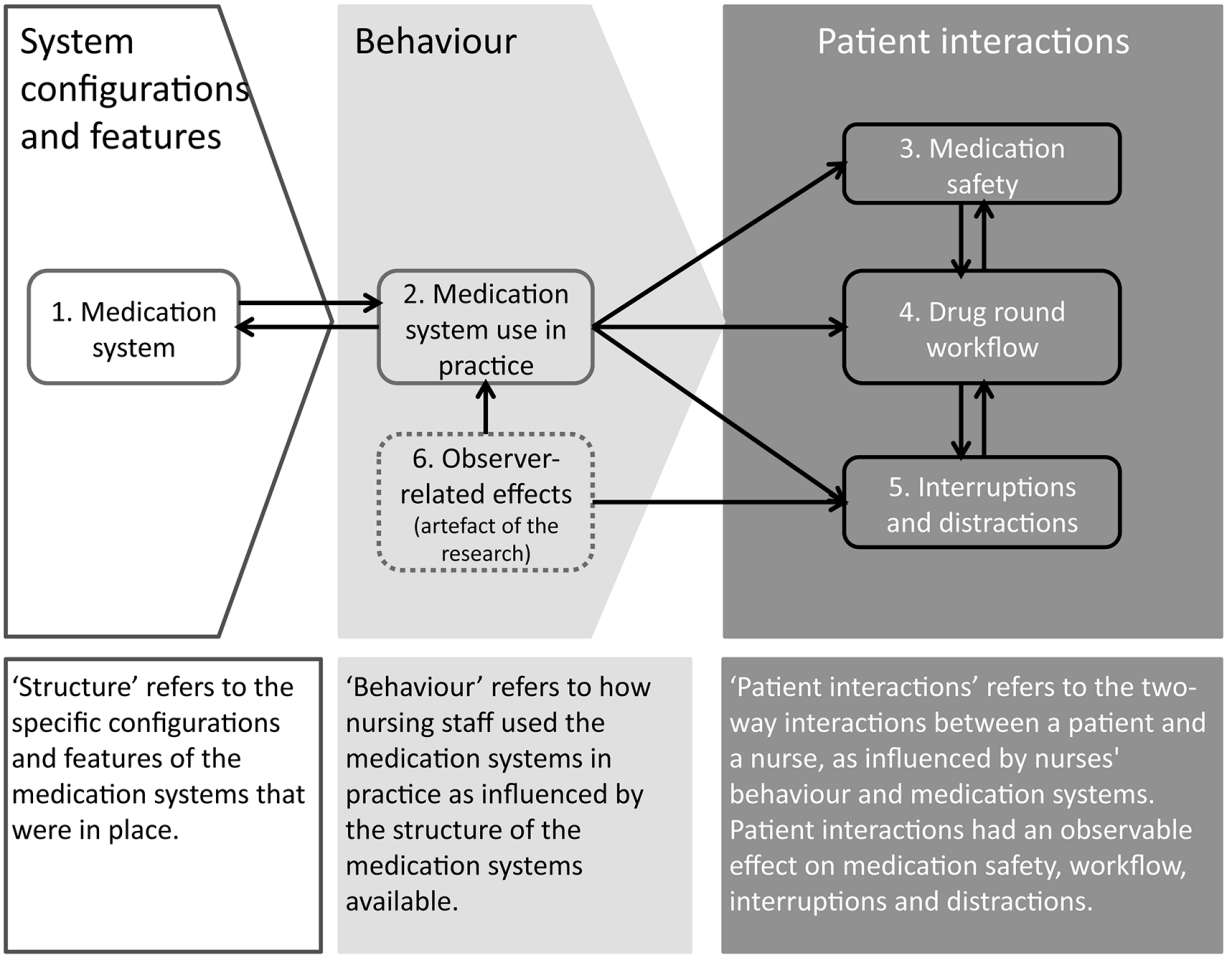
Conceptual overview of thematic factors that influence medication administration errors, workflow, interruptions and distractions associated with the hospital medication administration process. There are three over-arching interlinked themes: structure, behaviour, and patient interactions that encompass the six main areas (numbered). Arrows indicate the direction of influence between areas. Dotted lines indicate the presence of the observer as an artefact of the research directly on nursing staff behaviour, and on interruptions and distractions.

We identified three inter-related themes that encompassed the five main areas in our initial thematic framework; a sixth, ‘observer-related effects’, was added to reflect actual and potential effects of having an observer on both nurse and patient behaviour (Fig 1). The three inter-related themes were: (1) structure—related to configurations and features of the medication systems, (2) behaviour—referring to different types of nursing staff behaviour, and (3) patient interactions—referring to the two-way interaction between a nurse and a patient. Structure was the foundational theme that affected different types of nurse behaviour, which in turn, incited different types of patient interactions; each comprised components that exerted a positive and/or negative impact on medication safety, drug round workflow, interruptions and distractions.

### ‘Structure’—system configurations and features

**Fig 2:**
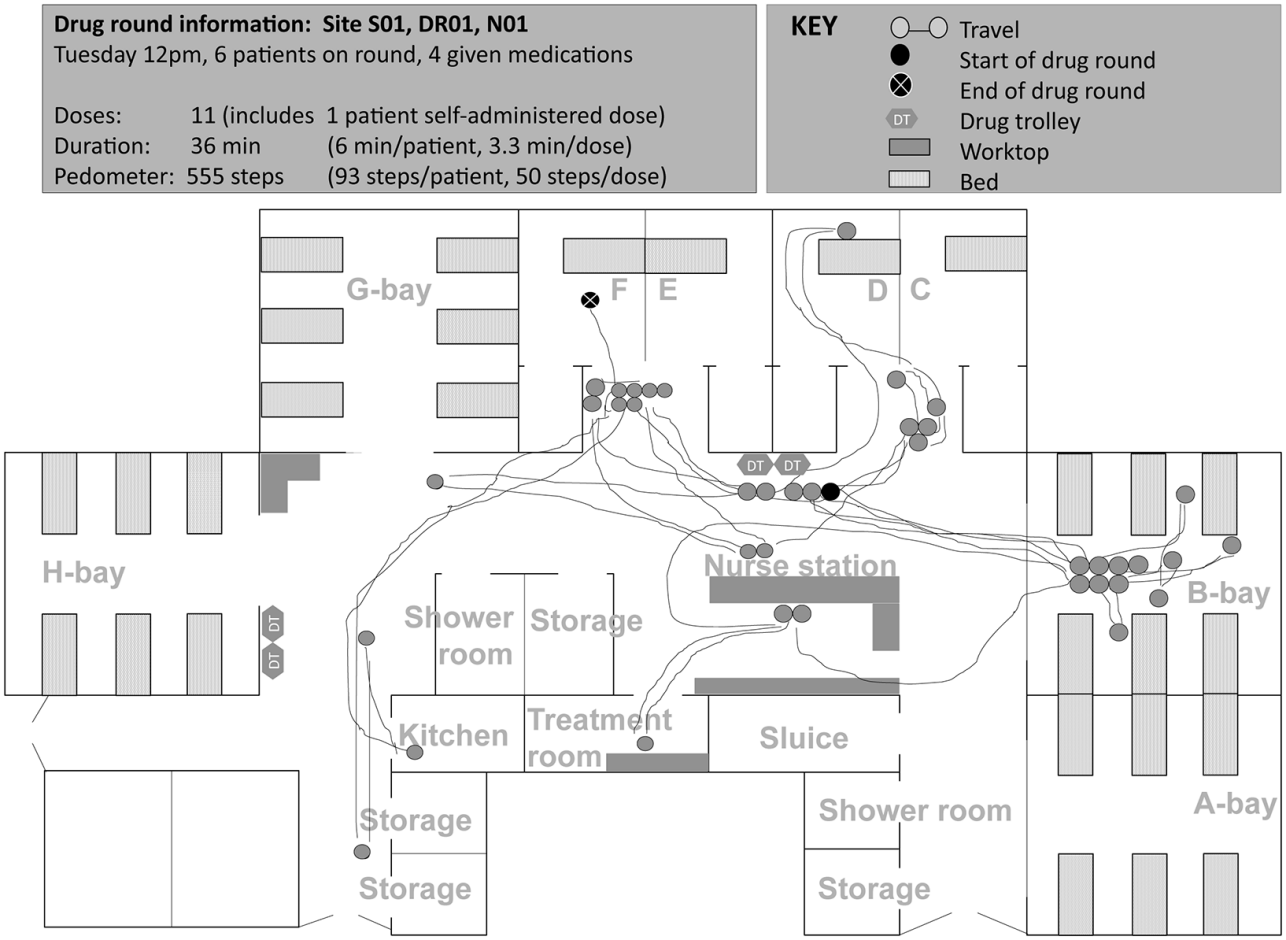
Spaghetti diagram showing non-linear travel by one nurse (qualified 2 years, 1 year experience on study ward) during a noon drug round at site A (map of ward not drawn to scale). Nurse started the drug round by wheeling the drug trolley from opposite the nurse station to side room D. Nurse went to another drug trolley located near G bay (3 times): once each to find medication, a tablet cutter and a plastic medication cup. Nurse also walked and attended to a patient other than the patient she was preparing medications for during the drug round (2 times), to the nurse station to look for a paper drug chart (2), to the kitchen to retrieve nutritional supplement (1), and to help another nurse to exit the ward (1). S01, site code; DR001, drug round code; N01, nurse code. Letters refer to ward bay areas.

**Fig 3:**
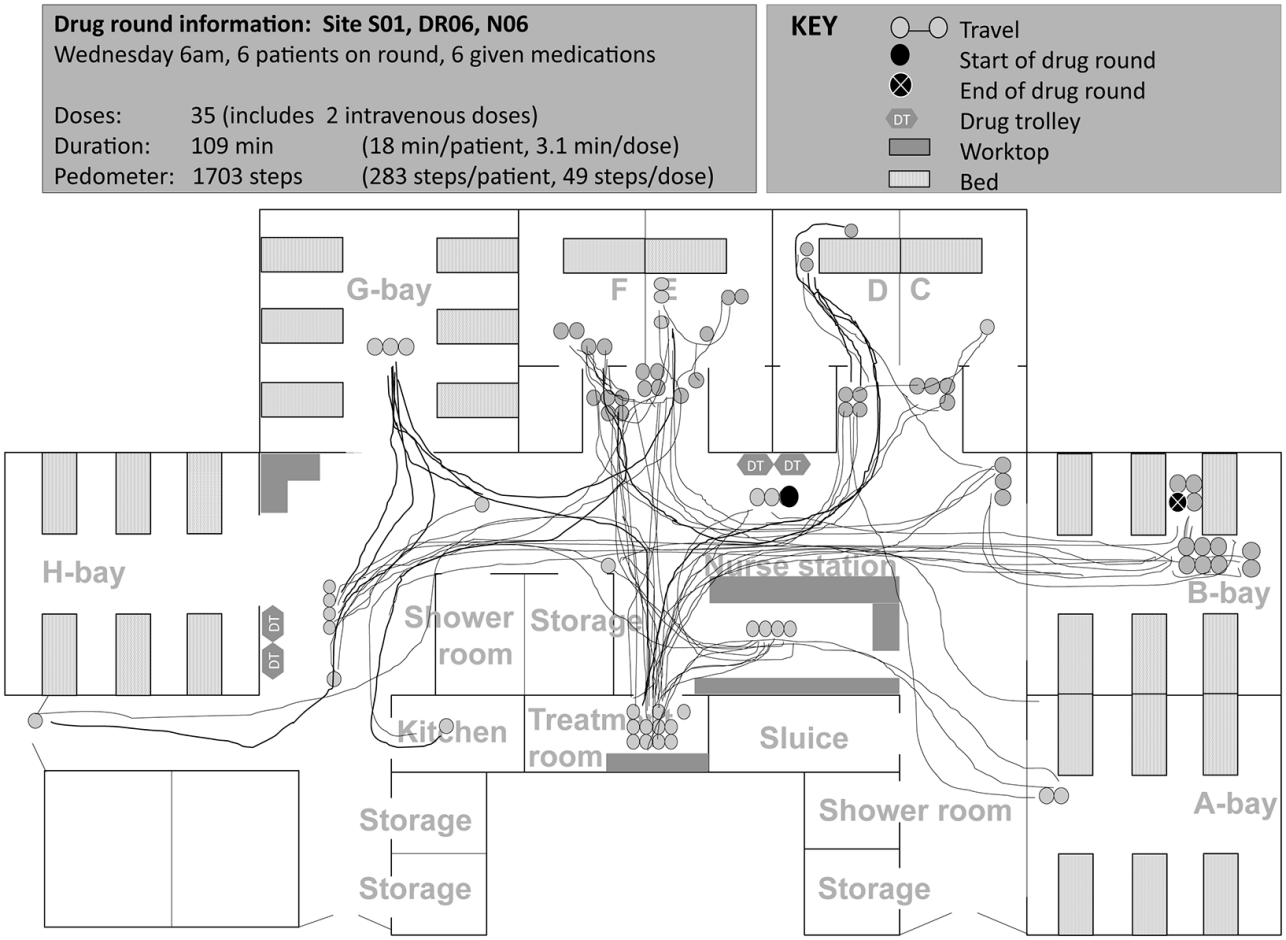
Spaghetti diagram showing non-linear travel by one nurse (bank staff) during a morning drug round at site A (map of ward not drawn to scale). Nurse started the drug round by wheeling the drug trolley from opposite the nurse station to side room D. Nurse went to the treatment room 11 times during the drug round: to look for medicines in the stock cupboard (2 times),to prepare medications for intravenous administration (5), to look for the drug chart (1) and to access the medicines fridge (3). During the drug round, the nurse also travelled to locations other than between the drug trolley and patients’ bedside: another drug trolley to look for medicines (4 times), nurse station to look for drug chart (1), nurse station to look for keys (2), kitchen to retrieve nutritional supplement (1), to another nurse to provide handover of patients (2), and to the ward next door to look for medicine (1). S01, site code; DR006, drug round code; N06, nurse code. Letters refer to ward bay areas.

**Fig 4:**
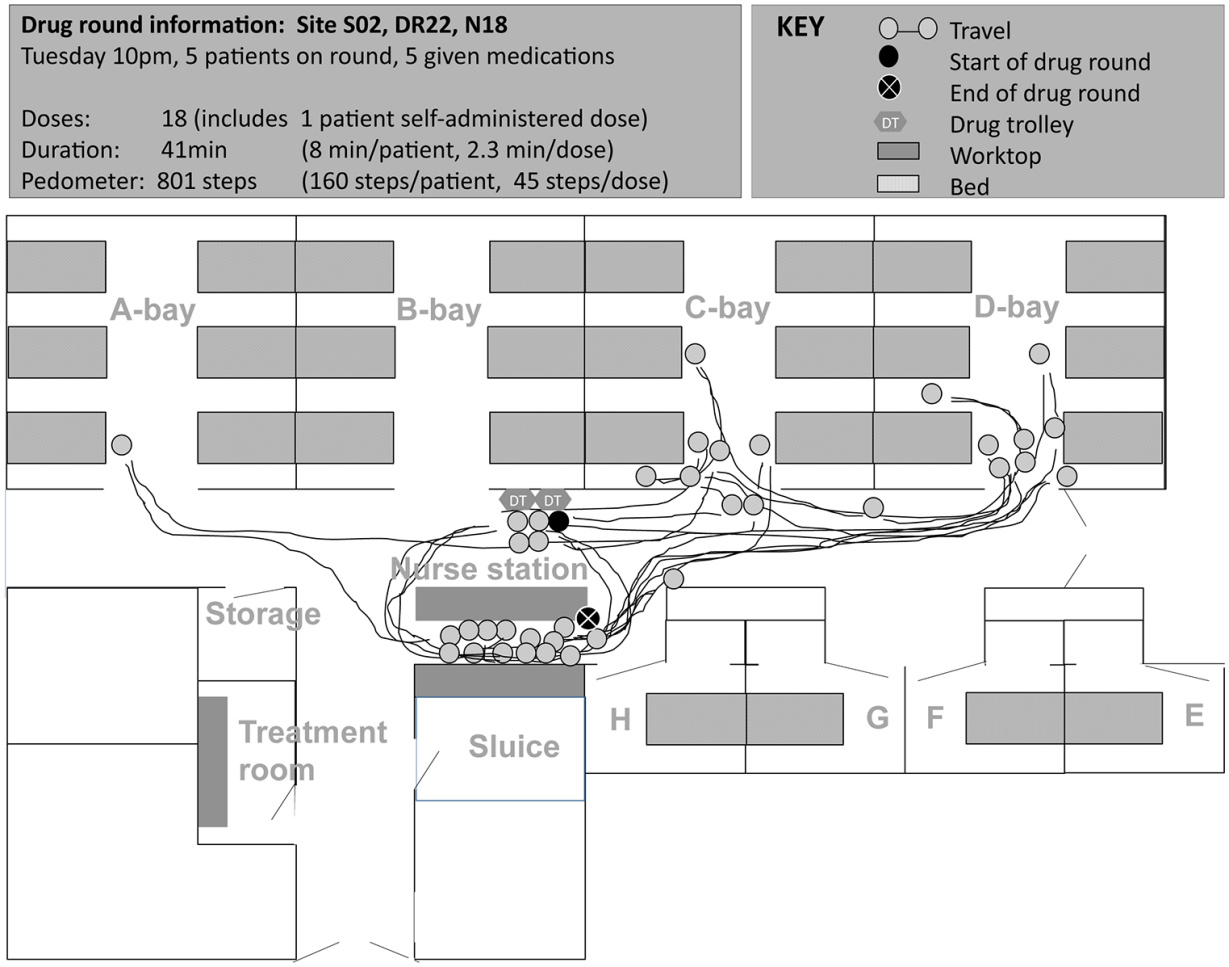
Spaghetti diagram showing non-linear travel by one nurse during night-time drug round at site B (map of ward not drawn to scale). Nurse started the drug round by logging on to the tablet computer next to the drug trolleys at 21:05, placed tablet computer on drug trolley and wheeled it to each patient starting in C-bay. Nurse went to the nurse base station area 13 times during the drug round: to look for master key to patient’s bedside medication locker (2 times), to look for medicines in stock cupboard (4), to access desktop computer to view and/or sign patient medication orders (5), to take a telephone call (1), and to prepare from the controlled drugs cupboard (2). Nurse ended the drug round at the nurse base station double checking on the electronic prescribing and medication administration system that all the relevant doses had been signed. S02, site code; DR022, drug round code; N18, nurse code. Letters refer to ward bay areas.

**Fig 5:**
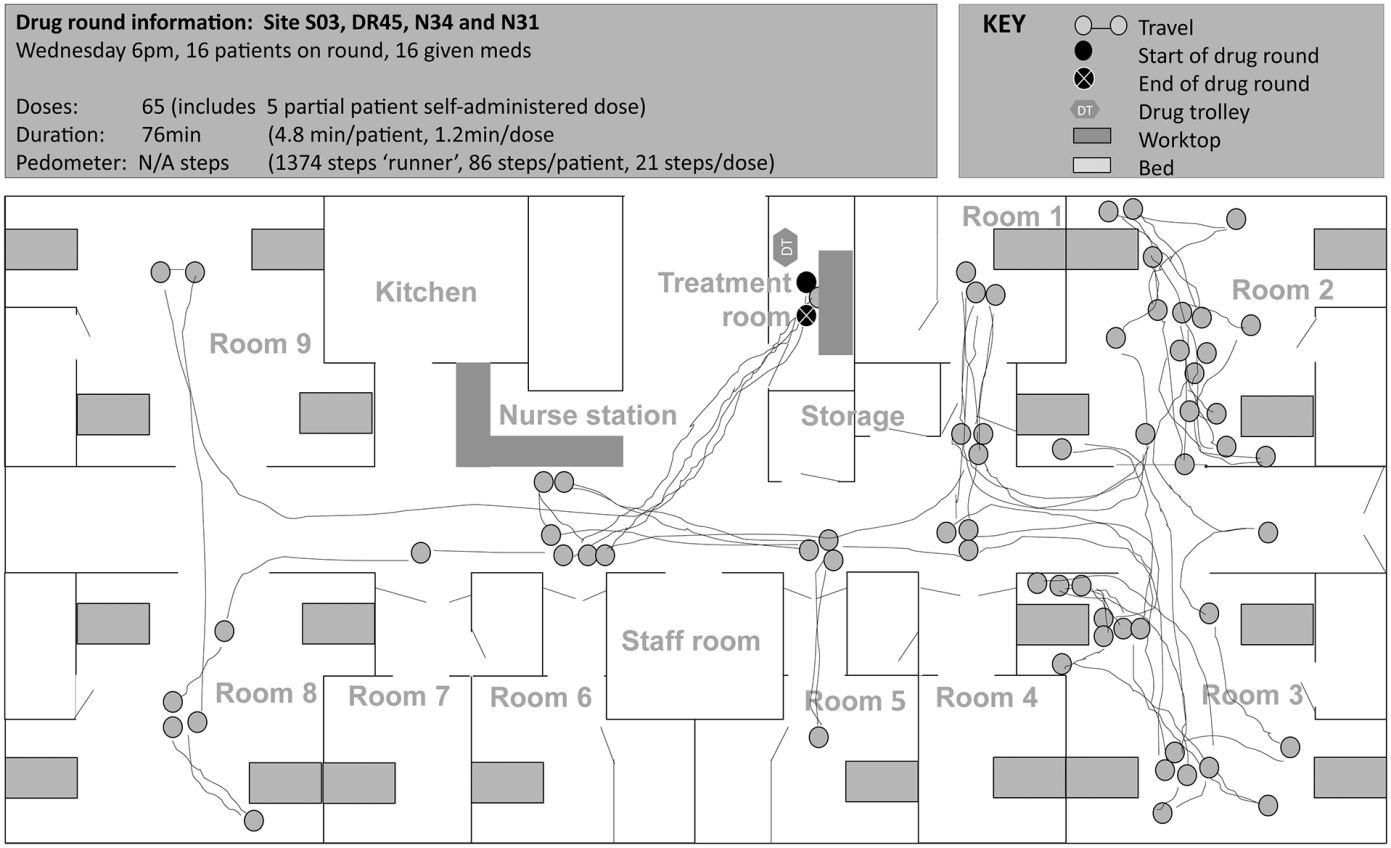
Spaghetti diagram showing changes in travel pattern of one nurse during a ‘two-nurse’ evening drug round at site C (map of ward not drawn to scale). At site C, two nurses typically worked together on the drug round to administer medications to all patients; one nurse ‘caller’ and one nurse ‘runner’. The diagram shows the path of travel by the nurse ‘caller’ who initially stayed with the drug trolley: she used the laptop attached to the drug trolley to access the patient’s electronic medication administration record, called out doses to the ‘runner’ to retrieve medications from the bedside medication locker and prepared some doses from the drug trolley. After preparing medicines for the patient in room 6, the nurse caller went ‘ahead’ while the nurse runner remained to administer the doses; this process was repeated whenever a patient required assistance to take the medicines and led to a ‘single-nurse’ drug round for parts of the remaining round. During the drug round, the nurse caller went to the nurse base station twice (to retrieve a patient‘s folder to check oxygen saturation and to retrieve another patient’s folder for paper warfarin medication order) and treatment room once (to retrieve medication from the fridge). S03, site code; DR045, drug round code; N34 and N31, nurse codes.

Specific configurations (location and arrangement of human and material resources) and features (characteristics, interpretability, and pre-conditions for use) of structure-related aspects [37] of the medication system acted as a physical constraint on some drug round tasks. Such structure-related aspects increased medication safety in some cases, but contributed to interruptions, distractions, impaired workflow, and medication problems in others (Table 2, Figs 2 to 5).

**Table 2:** Examples of system configurations and features, and their potential positive and negative effects on medication safety, workflow, interruptions and distractions.

Potential positive aspects of system configurations and features	Potential negative aspects of system configurations and features
Facilitating medication preparation next to drug chart/EPMA system (1) A desktop computer was near the stock cupboard for oral medicines, thus allowing nursing staff to check the EMAR while preparing medicines that may not be available from the drug trolley; (2) The patient bedside medication locker was a removable drawer which could be moved to an alternative area while preparing medicines (for example, if there was limited space at the locker to place the drug chart or mobile EPMA device, or if more than one drug was required from the bedside medication locker).	Practicalities of the drug chart/EPMA system (1)Drug administration codes for ‘patient refused’ and ‘patient did not require’ were used interchangeably Reported unreliability of tablet computer devices and font size too small on laptop led to nurses reporting a preference for using the desktop computer on some drug rounds. This meant that the EMAR was sometimes not used at the patient’s bedside or at the drug preparation location (e.g. treatment room); (2) Password and training required to use EPMA system, therefore EPMA could not be used by agency staff. Instead, regular nursing staff printed out medication administration records for agency staff and transcribed medication administration documentation on to the EPMA system after each drug round (signature on EPMA system was of the transcribing nurse).
Facilitating medication retrieval during drug round (1) Some patients kept their bedside medications together in a box which seemed to make it easier for nursing staff to find medications not stored in the bedside medication locker, for example, creams and inhalers; (2) Medications in the drug trolley were arranged such that the front (rather than the side) of most packs were facing the nurse to aid identification: (3) Drug trolley was kept in the treatment room and was often replenished immediately prior to and/or after the drug round; (4) Drug trolley was kept in the treatment room which also had a fridge; fridge items were placed in the drug trolley prior to starting the drug round.	Travel (1)Not all the medications or equipment (drug charts, keys, paper/plastic medicine cups) required during the drug rounds were available at the patient’s bedside. Some may be temporarily placed elsewhere but others such as infusion pump equipment were located in a separate room some distance away from the patient’s bedside and potentially increased travel for nurses; (2) The day room was located some distance away from the patient bed areas which was a particular problem on one ward as some patients were mobile and were often in the day room during drug rounds; thus potentially increased travel and opportunities for interruptions.
Reducing interruptions and distractions (1) Ward staff developed a standard form for documenting medication-related tasks that required follow-up after the drug round; (2) Ward staff placed a ‘ward screen’ at the entrance of a bay in which patients were being washed, this discouraged interruptions to anyone inside the bay including interruptions to nurses who were carrying out the drug round at the same time; (3) Nurse checked EPMA at the nurse station prior to starting lunchtime drug round for doses that were due. Nurse expected very few doses and did not use drug trolley on the drug round but prepared medications at the nurse station from the stock cupboard.	Medication security and accessibility (1) A few medicines (for example, nebules and pre-filled syringes) were sometimes kept on the shelf at the bottom of the drug trolley in addition to inside the drug trolley which was accessible to passers-by; (2) Some frequently used IV drugs (e.g., paracetamol [acetaminophen] and metronidazole) were stored on high shelves which made them difficult to retrieve; (3) Some patient bedside medication lockers were positioned so that the locker opened towards the bed to facilitate patient self-administration (rather than towards the nurse opening it) which made it more difficult for the nurse to access the contents when the patient was not self-medicating; (4) Nurses had to stoop to open patient bedside medication lockers.

Abbreviations: EMAR, electronic medication administration record; EPMA system, electronic prescribing and medication administration system; HCA, health care assistant; HCP, health care professional; IV, intravenous.

Both potentially ‘positive’ and ‘negative’ system configurations and features for medication safety were identified on each study ward; these were grouped under broad headings as in Table 2. Nurses rarely commented on the benefits of existing medication systems and processes on medication safety, workflow, interruptions or distractions; however nurses did report perceived negative aspects as areas for improvement. In general, few nurses sought to resolve underlying problems during the periods observed. In a number of cases, individuals seemed to have accepted these and worked around the problem:

*Nurse sometimes likes to put two drug trolleys together so she can prepare the medicines more easily [implied medications were not always available from one drug trolley]*.

(Site A, comment documented during a drug round. Nurse had one year of experience on the ward)

*Nurse preferred to use the tablet computer over the computer on wheels (COW) as she found the mouse pad tricky to use on the COW*. *However*, *she preferred to sign for medication administrations at the desktop as the tablet computer was too small*.

(Site B, comment documented during a drug round. Nurse had over seven years of experience on the ward)

Based on individual feedback and observations, the type of action taken to manage perceived medication system related problems or inefficiencies seemed to partly depend on individual behaviour types, which are next described.

### ‘Behaviour’—types of behaviour among nursing staff

As illustrated in Figs 2 to 5, medication administration was not a linear process; nurses encountered a number of tasks which took them to locations other than the patient’s bedside. Observed variation between individual approaches to drug round tasks, even on the same ward, suggests that workflow was not only influenced by structure-related configurations and features, but also by behaviours that partly depended on the individual (‘inherent behaviour’) and on the immediate environment in which medications were given (‘situational behaviour’).

**Fig 6:**
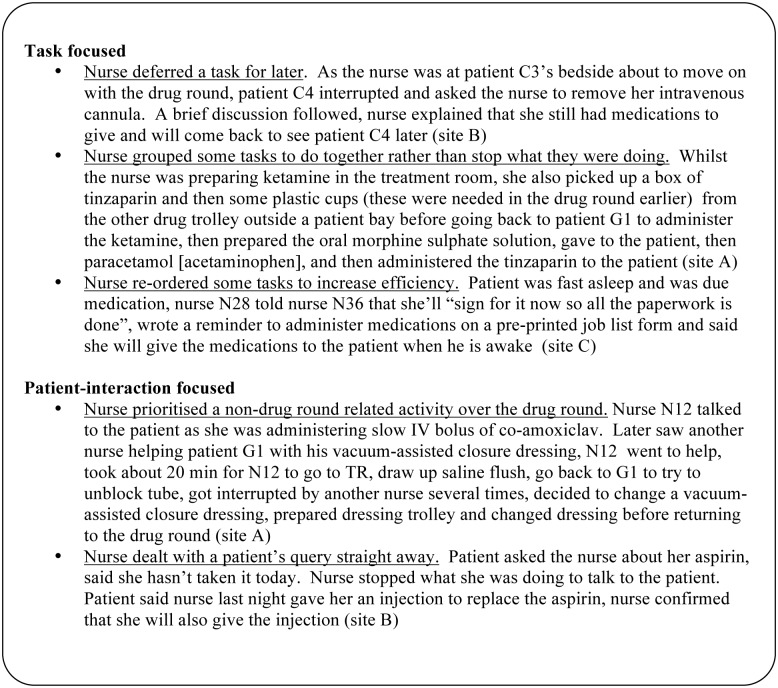
Examples of inherent behavioural tendencies and associated influences on how systems were utilized, and how medication administration related problems, interruptions, distractions, and workflow were managed.

Broadly, nurses appeared to have an inherent tendency to be either primarily ‘task focused’ (main goal of drug round appeared to be administer drugs as efficiently as possible), or ‘patient-interaction focused’ (drug round appeared to be an opportunity for the nurse to interact with their patients in addition to administering medications). Excluding urgent tasks, task-focused individuals generally used a streamlined workflow and carried out few non-medication administration related tasks during the drug round; when the need for such tasks was identified during the round, the nurse either deferred the task to the end of the round, or carried out the task when another task took the nurse to a convenient location to carry out multiple tasks together (Fig 6). By contrast, patient-interaction focused individuals adopted a relatively less streamlined workflow, and appeared to encourage communication with patients and/or other staff during the round; the patient-interaction focused individuals either multi-tasked, carried out the non-medication administration related task shortly after they completed the primary task, or stopped the primary task to carry out the non-medication administration related task.

**Fig 7:**
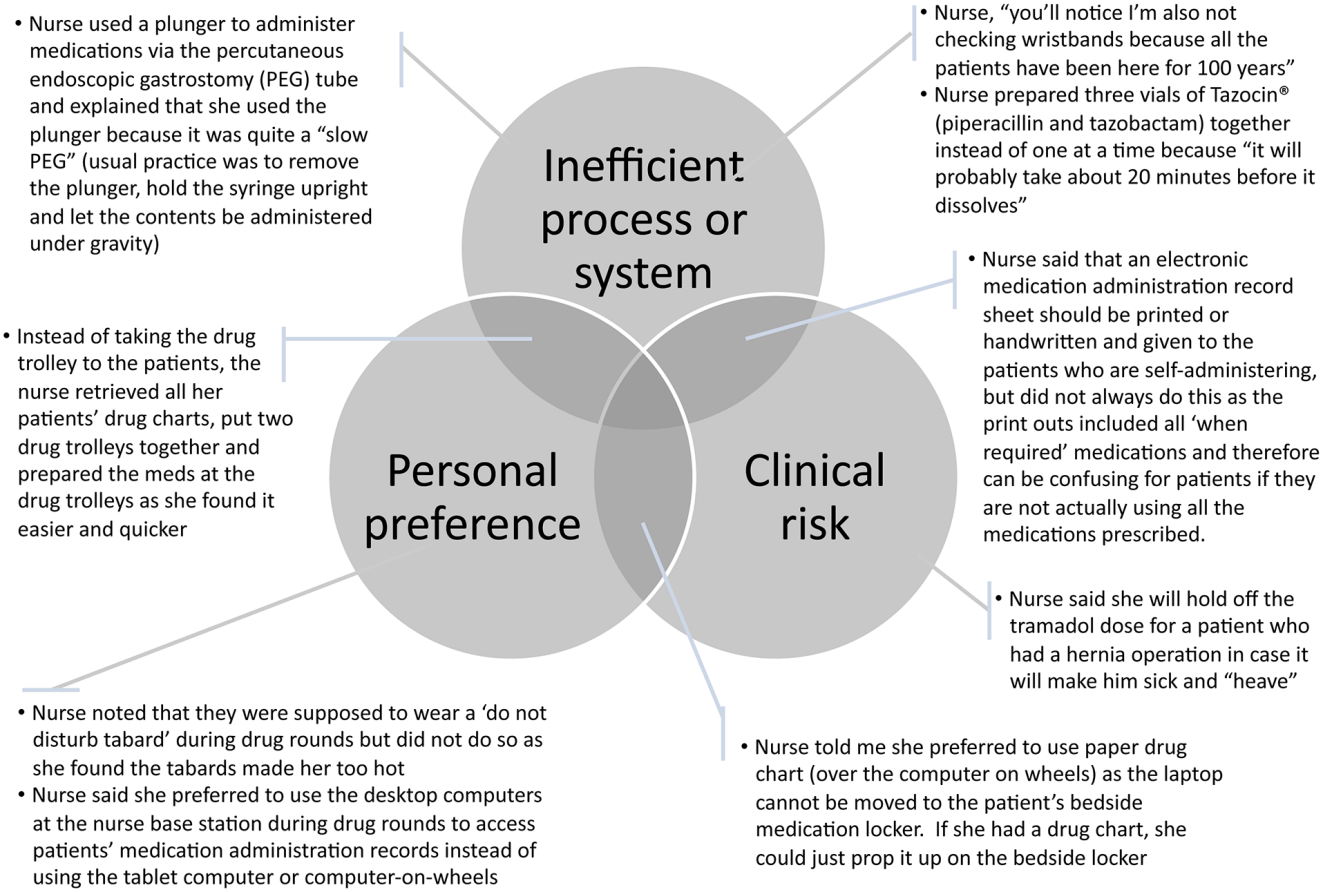
Three main overlapping reasons for intentional ‘alternative’ practices identified from nurses’ feedback.

Additionally, a number of self-reported intentional ‘alternative’ practices (‘non-conforming behaviour’) from ward routines and trust policies were identified: some examples are presented in Fig 7. We identified three main overlapping reasons for non-conformance: perceived inefficient process or system, clinical risk, and personal preference. The resulting action may or may not violate policy.

Overall, all behaviour types had the potential to either increase or decrease medication safety. Sometimes the alternative practice observed was part of an established tried-and-tested routine for the individual; at other times it was more spontaneous. In general, the behaviour types exhibited were not fixed; individuals appeared to shift from one to another, depending on the needs of the patient, the medication system being used at the time, the task being carried out, and other situational circumstances.

**Fig 8:**
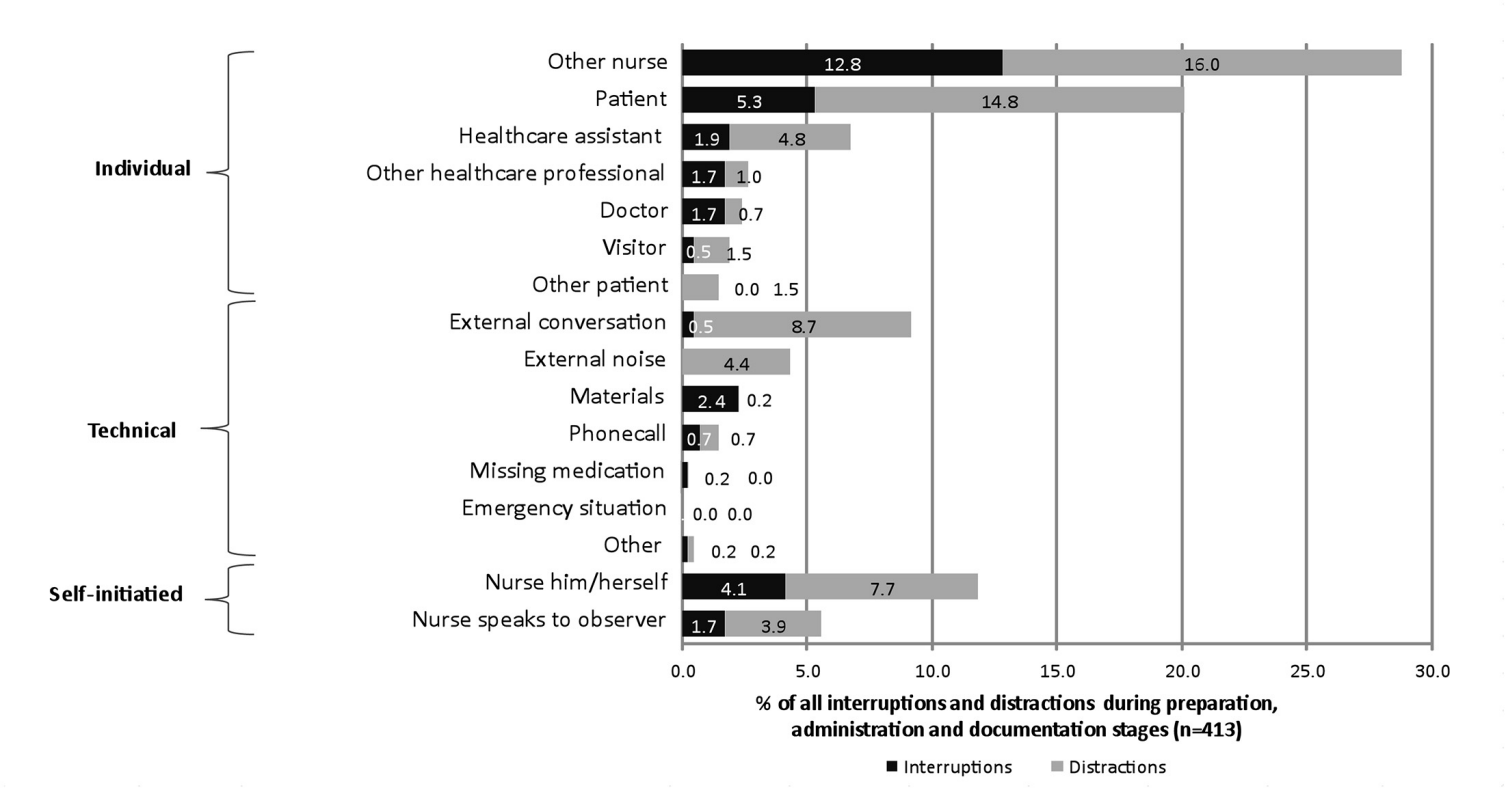
Sources of interruptions and distractions during drug rounds percentage of a total of 413 interruptions and distractions observed at the preparation, administration, and documentation stages of drug rounds). Median 5.5 interruptions per drug round hour, range 0 to 24; median 9.6 distractions per drug round hour, range 0 to 30; median 15.5 interruptions and distractions combined per drug round hour.

Given that the nurse him/herself was the third most common source of interruptions and distractions (Fig 8), it is likely that individuals’ inherent tendencies may influence the potential for MAEs. However, the ‘direction’ of influence (positive or negative) on drug round workflow and MAEs depended on the medication systems being used and the task being carried out at the time. By contrast, ‘other nurses’ were the most common source of interruptions and distractions to the nurse on the drug round. Observations suggest that these interruptions and distractions were frequently made by those who themselves were also involved in medication administration around the same time; multiple nurses administered medications simultaneously to their own individual patients on two study wards and therefore the same medication system problems were potentially affecting the nurses at the same time. The percentages of other sources of interruptions and distractions are summarized in Fig 8; the presence of the observer had a measurable effect on the number of interruptions and distractions experienced by the nurse. However, the overall percentage of observer-related interruptions and distractions was considerably less than those from patients despite the observer’s continued presence.

### Patient interactions

As depicted in Fig 1, interactions between patients and nursing staff resulted in an observable effect on medication safety and drug round workflow. Patients were the second most common source of interruptions and distractions during drug rounds (Fig 8) and therefore potentially contributed to reduced medication safety. However, some nurse-patient interactions potentially increased safety. Specifically, we found three manifestations of the patient being a defence against medication errors: (1) patients as an active resource of information (volunteering information about their medicines without prompting), (2) patients as a passive resource of information (providing information about their medicines when asked or prompted), and (3) patients acting as a ‘double-checker’ with the intention to check the medication being prepared or administered (Table 3).

**Table 3:** Three observed manifestations of the patient as a defence against medication errors.

Description	Examples
Patients as an active resource of information (volunteered information) about their medicines	• Patient highlighted discrepancy in pregabalin dose, told the nurse it should be 250mg twice a day, but it was prescribed as 100mg twice a day, nurse documented this and talked to patient about changes in medications. Later it was confirmed that the wrong dose had been prescribed (site C).
	• Patient told the nurse that she (the patient) could not break up the cocodamol [contains paracetamol and codeine] and therefore did not take the dose that was given to her in the previous drug round. The dose had been signed as administered but was not actually taken. Nurse was aware, helped patient crush tablets by using two spoons (could not find tablet crusher on the ward) (site B).
Patients as a passive resource of information (provided information when asked or prompted) about their medicines	• Nurse noticed on the drug chart that the patient had not received tinzaparin recently (there were two doses crossed off and one blank administration box), she asked the patient "do you know of any reason why you haven't been given the tinzaparin?" "I get it on dialysis" replied the patient. Tinzaparin had been prescribed for once daily administration and there was no documentation on the drug chart to indicate that the patient was to receive this on dialysis days only (site A).
	• Nurse told the patient what she was giving (included naproxen and omeprazole); patient explained he takes both at night: “only take it at night” “not morning?” “only take it at night” “ah they prescribed it for this morning…..I don’t know why [they] prescribed it for morning” explained to patient that she did not give these last night and so patient took the medications at the morning drug round (site B).
	• Medication order did not specify which eye(s) to which the eye drops were to be applied. Nurse asked the patient, "your eye drops, do we do it for you or you do it?" "You do it" "Is it both of the eyes?" Patient confirmed it was for the right eye, nurse administered it to the patient’s right eye (site A).
Patient acted as a double-checker during drug rounds	• Metformin dose prescribed was 500mg- 1g three times a day on the drug chart and the prescriber had written “1g OM” (meaning once daily in the morning) in additional section of chart for metformin. Nurse had prepared 500mg and given to patient during a morning drug round but later corrected it when prompted by the patient and gave 1g in total (site A).
	• Nurse went straight to the patient’s bedside medication locker to retrieve the patient’s own gliclazide, omeprazole, metronidazole and pioglitazone. During this time, the patient asked “is it metformin?” Patient told the nurse that the metformin was in the same packet as the gliclazide (site B).

While patient interactions primarily related to relationships between nursing staff and the patient, a number of system-related influences on these relationships were also observed. For example, nurses typically did not take the computer on wheels (sites B and C) or drug trolley (all sites) into patient side rooms (single-bed), and often relied on their memory and/or brought medications out of the room to prepare doses, thus potentially reducing patient involvement. Patient involvement was important not only as a potential defence barrier for MAEs but also to optimize their treatment. The dose omission rate due for clinical reasons, such as a patient declining to take tramadol as it made them feel sick and they were not in any pain, was 11.4% of 458 OEs, many of which were the result of direct nurse-patient interaction during the drug round.

## Discussion

This is the first study to combine qualitative and quantitative methods to explore how nurses administer medications safely to hospital inpatients despite various challenges of the ward environment. Variations in hospital ward medication systems in English NHS hospitals exist [18,27] but we identified much more subtle variations than previously reported. Overall, medication administration is not a linear process, and we identified three inter-related themes that acted as both facilitators and barriers to safe medication administration. The first relates to specific configurations and features of the ward-based medication system (theme 1). This in turn can influence nursing staff behaviour (theme 2) in terms of workflow, how nurses manage interruptions and distractions, and how they interact with patients (theme 3). Based on our findings, a number of system-related nurse behaviour types were identified. Importantly, nurses appeared to have a general inherent tendency to be either primarily ‘task focused’ (main goal of drug round was to administer medications as efficiently as possible), or ‘patient-interaction focused’ (drug round was an opportunity for the nurse to interact with their patients in addition to administering medications) during the drug round. Both types of behaviour had the potential to increase medication safety in different ways: task focused behaviour may lead to a more streamlined and efficient workflow thereby minimizing interruptions and distractions that potentially contribute to MAEs [16], while patient-interaction focused behaviour may lead to increased patient involvement with their medication thereby better enabling the patient to act as a defence against errors. A focus on alternative practices also led to the identification of three overlapping causes for intentional non-conformance. Analysis of alternative practice behaviour was based on nurses’ feedback during the observations and was therefore primarily associated with structure-based inefficiencies. Other unreported reasons for alternative practices were not explored. Nonetheless, our findings support previous research which identified that “workarounds can [both] subvert and augment patient safety” [25]. Furthermore, by studying nurse behaviour types, our research suggests that potential latent conditions for MAEs, analogous to the ‘resident pathogens’ described by Reason [13], can be identified by examining non-conformance with typical local practices.

Consistent with the literature, patients sometimes acted as a defence barrier to medication error [38]. We identified and conceptualized three ways in which patients acted as a defence, emphasizing the importance of patient involvement in their medicines even in hospital. The challenge is identifying how this can best be facilitated, while recognizing how this is likely to change during different stages of the inpatient stay.

### Implications for practice

In the UK, the high-profile Francis Report [6–8] highlighted inadequate nurse staffing (numbers, skill mix, knowledge and experience) as a major patient safety concern in one NHS trust. However, our study highlights additional complex issues; it provides a timely insight into specific challenges of the work environment in three different wards and how nurses worked within them to administer medications safely.

First, we have identified practical examples of resource optimization to increase medication safety which may be used as a platform for further discussion within individual healthcare organizations. Intuitive adoption of safe medication practice behaviours may be facilitated by optimizing system configurations and features [39]. Optimizing ward-based medication systems to streamline workflow may therefore be an effective and cost-neutral way to increase medication safety; this could include simple steps to ensure that all relevant medication, documentation and equipment is available in one place when needed during medication administration (Table 2). However, how can potentially suboptimal processes and systems be identified? Our research suggests that some nurses express their identification of known system-based problems by developing routine alternative practices or workarounds. It is therefore important that healthcare organizations engage effectively with ward staff to ‘tap into’ their tacit knowledge of system problems. Direct observation is an important research method that allows some of this tacit knowledge to be identified; however this approach is likely to be too time-consuming to be of practical use on a regular basis. Instead, the use of ‘soft intelligence’, for instance information gathered from conversations, observations and experiences, incorporated into regular routine multi-professional walkarounds may be more practical [40]. As highlighted by Berwick [9] in his report on the Francis Inquiry, “most healthcare organizations have very little capacity to analyse, monitor, or learn from safety and quality information”, and therefore more inventive methods are needed. Hard information, such as objective and quantitative data has long been used in the NHS and other healthcare institutions as an indicator of performance. However research suggests the use of such information in isolation can be misleading [41]. Evidence suggests that a combination of hard and soft information can be complementary and allow better triangulation of findings on which to base decisions; the risk of using soft information only is that decisions may be based on out of date anecdotes rather the current experience [40,41].

Second, nurses are highly adaptable healthcare professionals who need to balance conflicting priorities and demands during drug rounds. While medication administration is one of many nursing tasks, research suggests that senior clinical nursing staff are concerned that their staff are becoming overly task-focused in general and providing less patient-centred care [42]. In our study, we found both task focused and patient-interaction focused behaviour to have different potential benefits. Our conceptual analysis of the two types of inherent nurse behaviour may therefore offer a way to facilitate behaviour change in nursing staff, for example, by helping task focused nurses to better see the importance and benefits of a patient-interaction focused approach, and vice versa for primarily patient-interaction focused nurses. This could be based on a supportive peer-review process in which staff periodically observe each other’s medication administration practices and consider the advantages and disadvantages of different approaches. To further support the development of beneficial nurse-patient interactions, we suggest efforts should also be focused on supporting nurses to manage interruptions and distractions, some of which are potentially important and beneficial [43], rather than avoiding them completely, as advocated elsewhere [16,31]. Further work should focus on characterising and differentiating positive and negative interruptions, identifying strategies to prevent avoidable and non-urgent ‘delayable’ interruptions, and supporting nursing staff in addressing important beneficial interruptions in an appropriate manner. In advocating an increase in patient involvement with their medications (beyond self-administration) to enhance medication safety, it is also important to consider potential unintended consequences of actual or perceived shifting of responsibility to the patient.

Finally, our research exemplified the potential benefits of the observational approach to ‘looking’ and ‘seeing’ practices in natural settings to identify safe practices that are not always recognized or reported by staff. Thus, there is a need to use observational methods strategically and share the findings across healthcare organisations to maximize their utility and increase patient safety.

### Comparison of quantitative findings with previous research

The MAE rate for non-IV OEs identified in the present study (2.7%; 95% CI 1.2–4.2%) was lower than previously reported in a systematic literature review of UK studies using similar observational methods (5.6%; 95% CI 4.6–6.7%) [2]. The lower MAE rate may be partly due to the relatively restricted range of medications used on the study wards; a large proportion of patients were either admitted electively, transferred from another ward or undergoing rehabilitation. The present study identified a median rate of 5.5 interruptions per drug round hour and 9.6 distractions per drug round hour. The former is similar to the 6.7 interruptions per hour reported in the literature review of Biron et al [31], based on a total of 2,622 interruptions observed in 14 studies across a range of acute care settings. However, the definition of an interruption varied between studies and it was unclear whether or not the interruption rates reported by Biron et al [31] included ‘distractions’, which we defined and reported separately.

### Strengths and limitations

Strengths of our research were (1) inclusion of multiple wards that used distinctly different medication systems to reflect some of the diversity of practices within the English NHS, (2) using an ethnographic approach, rather than self-report, to identify ‘real-world’ practices, (3) using a mixed-methods approach to triangulate our findings, and (4) reporting quantitative data based on established methods and definitions to facilitate comparison with previous studies. Furthermore, informal feedback from nursing staff about the observation experience was generally positive and the presence of the observer was not perceived to be a problem. Staff seemed generally quite open about their opinions of the systems relating to medication administration and provided invaluable additional insights into their rationale for approaches taken during drug rounds.

Sociotechnical interactions are complex; there are multiple interconnecting systems and processes that are not always apparent. Limitations of our research were that (1) we did not explore the wider impact of system optimization on the work processes of other health care professionals, (2) we did not explore other individual nurse or environmental factors, such as age and qualifications, or noise and lighting, that may be associated with nursing errors and efficiency [44], (3) our study was not powered to detect quantitative differences between wards, such as the effect of paper versus electronic systems on medication safety, or the relationship between structural factors and errors, distractions or interruptions—our findings may be used to design quantitative studies to explore these issues, (4) despite efforts made to maintain a balance between objectivity and subjectivity (e.g. encouraging feedback from nursing staff and collection of objective data to support our interpretation), data were collected by one researcher to facilitate the formative nature of ethnographic research and therefore may be limited to some degree by the beliefs and experience of the researcher. Furthermore, findings from the interruptions and distractions recorded in the current study indicate the presence of the observer had a measurable influence on nurse behaviour during drug rounds. Thus, the observer may have influenced MAE rates in a positive way through the Hawthorne effect. However, it is also possible that the observer increased the risk of an MAE by being a distraction to the nurse. While previous studies suggest that there is no effect of observation on MAE rates provided the observer is discreet, non-judgmental, and tactful [45,46], our study was not designed to quantify this further. We suggest future observational studies of MAEs consider documenting the frequency of participant-observer interactions.

## Conclusion

Overall, a number of subtle structural variations in available resources appear to influence individual nurse behaviour and patient interactions, with some notable positive and negative unintentional consequences on medication safety. Our findings suggests that efforts to reduce MAEs be focused on three main areas (1) optimization of ward-based medication systems, (2) supporting nurses to manage interruptions and distractions, and (3) actively encouraging inpatient involvement with their medications where appropriate.
